# Nutritional quality of meals and snacks assessed by the Food Standards Agency nutrient profiling system in relation to overall diet quality, body mass index, and waist circumference in British adults

**DOI:** 10.1186/s12937-017-0283-0

**Published:** 2017-09-13

**Authors:** Kentaro Murakami

**Affiliations:** Interfaculty Initiative in Information Studies, University of Tokyo, Tokyo, 113 0033 Japan

**Keywords:** Meal, Snack, Diet quality, Obesity, Nutrient profiling system

## Abstract

**Background:**

Studies examining meal and snack eating behaviors in relation to overall diet and health markers are limited, at least partly because there is no definitive consensus about what constitutes a snack, a meal, or an eating occasion. This cross-sectional study examined how nutritional quality of meals and snacks is associated with overall diet quality, body mass index (BMI), and waist circumference.

**Methods:**

Based on 7-d weighed dietary record data, all eating occasions were divided into meals or snacks based on time (meals: 0600–1000, 1200–1500, and 1800–2100 h; snacks: others) or contribution to energy intake (EI) (meals: ≥15%; snacks: <15%) in 1451 British adults aged 19–64 years participating in the National Diet and Nutrition Survey. Nutritional quality of meals and snacks was assessed as the arithmetic EI-weighted means of the British Food Standards Agency (FSA) nutrient profiling system score of each food and beverage consumed, based on the contents of energy, saturated fatty acid, total sugar, sodium, fruits/vegetables/nuts, dietary fiber, and protein per 100 g.

**Results:**

Irrespective of the definition of meals and snacks, higher FSA scores (lower nutritional quality) of both meals and snacks were associated with unfavorable profiles of individual components of overall diet, including lower intakes of fruits/vegetables/nuts and higher intakes of biscuits/cakes/pastries, total fat, and saturated fatty acid. The FSA scores of meals and snacks were also inversely associated with overall diet quality assessed by the healthy diet indicator (regression coefficient (β) = −0.22 to −0.17 and −0.06 to −0.03, respectively) and Mediterranean diet score (β = −0.25 to −0.19 and −0.08 to −0.05, respectively) in both sexes (*P* ≤ 0.005). However, the associations were stronger for meals, mainly due to their larger contribution to total EI (64% to 84%). After adjustment for potential confounders, only the FSA score of snacks based on EI contribution was positively associated with BMI and waist circumference in women (*P* ≤ 0.005).

**Conclusions:**

Although lower nutritional quality of both meals and snacks assessed by the FSA score was associated with adverse profiles of overall diet quality (but not necessarily adiposity measures), stronger associations were observed for nutritional quality of meals.

## Background

Nutrition research has traditionally concentrated on the detailed examination of possible health roles and consequences of dietary components (foods, nutrients, or both) in isolation. However, the effects of individual foods and nutrients on health are usually difficult to estimate, given that they can be small [1]. Additionally, foods and thus nutrients are consumed in combination and their combined effects may be interactive or synergistic [2]. Further, because when eating, people mainly choose to combine foods in meals or snacks as per specific compositions [3, 4], there should be some patterns or characteristics in meals and snacks. Thus, understanding how nutritional quality of meals and snacks is associated with overall diet quality and health status (such as measures of body fatness) is important for the development of science-based recommendations of meals and snacks for consumers [5]. While Dietary Reference Intakes and other dietary recommendations are given on a per day basis, nutritional advice considering meals and snacks separately might also be easier and more practical for people to understand and follow dietary guidelines [5, 6].

Nevertheless, only a limited number of studies have been conducted to date to examine meal and snack eating behaviors in relation to overall diet and health markers [7–11], at least partly because there is no definitive consensus about what constitutes a snack, a meal, or an eating occasion [5]. While some researchers have relied on respondents’ self-identification of meals, snacks, or eating occasions [7–9, 12–18], others have attempted to use more objective criteria (based on clock time, energy content/contribution, or both) [8–10, 19–30]. An accurate distinction between meals and snacks is important, because they are hypothesized to have different effects on energy and nutrient intakes [10, 31, 32]. An understanding of the influence of different meal and snack definitions on the associations between nutritional quality of meals and snacks with overall diet quality and measures of body fatness may help establish consensus on the most appropriate research definition for meals and snacks [5]. Additionally, the associations of meal and snack intakes or patterns with measures of body fatness (as well as dietary intake) may be confounded by possible under-reporting of eating frequency (that is, meal and/or snack intake) concomitant with the under-reporting of energy intake (EI) particularly by obese or overweight subjects [33, 34].

Another important issue is a lack of established tools for assessing nutritional quality of meals and snacks [6]. In this context, the British Food Standards Agency (FSA) nutrient profiling system [35–37] may be a suitable or attractive choice. The FSA score of overall diet has been shown to be prospectively associated with certain health outcomes such as metabolic syndrome [37] and weight change [38]. One advantage of the FSA score is the fact that it is calculated based on the contents of individual components (i.e., energy, saturated fatty acid (SFA), total sugar, sodium, fruits/vegetables/nuts, dietary fiber, and protein) per 100 g of foods and beverages consumed and also is weighted for EI. Thus, it can provide a proportional measure of meal and snack quality in relation to EI.

The aim of this cross-sectional study in British adults was to examine how nutritional quality of meals and snacks assessed by the FSA nutrient profiling system is associated with overall diet quality, body mass index (BMI), and waist circumference (WC), by the use of different definitions of meals and snacks.

## Methods

### Survey design

This cross-sectional study was based on the National Diet and Nutrition Survey (NDNS): Adults Aged 19 to 64 Years. Details of the rationale, design, and methods of the survey have been described in detail elsewhere [39]. Briefly, the sample was selected using a multi-stage random probability design with postal sectors within mainland Great Britain as first stage units. Eligibility was defined as being aged 19–64 y and not pregnant or breast-feeding. One eligible adult per private household was selected at random. Data collection was conducted from July 2000 to June 2001.

### Anthropometric measurements

All anthropometric measurements were performed in duplicate by trained fieldworkers, and the mean value of two measurements was used in the analysis. Height (to the nearest 0.1 cm) and weight (to the nearest 0.1 kg) were measured while the subject was barefoot and wearing light clothes only. BMI (kg/m^2^) was calculated as weight (kg) divided by height (m) squared. WC was measured at the midpoint between the iliac crest and the lower rib (to the nearest 0.1 cm).

### Dietary assessment

Dietary data were collected by a 7-d weighed dietary record. A detailed description of the procedure has been published elsewhere [39, 40]. Briefly, each subject was supplied with a set of accurately calibrated Soenhle Quanta digital food scales and recording diaries. The subject was given written and verbal instructions by trained interviewers on how to weigh and record items in the diary. When weighing was not possible (e.g., eating out; 47% of total food items recorded), the subject was asked to record as much information as possible. Trained interviewers visited the household at least twice during the recording period and checked the completeness of food recording. All the collected diaries were checked by trained nutritionists in terms of coding, recorded weights, and descriptions of items consumed. Estimates of daily intake for foods, energy, and selected nutrients were calculated based on the Food Standards Agency nutrient databank [41], which is in turn based on McCance and Widdowson’s Composition of Foods series [42] and manufacturers’ data where applicable. For all dietary variables, mean values over 7 d were used in the analysis. Values of food and nutrient intake were energy-adjusted using the density method (i.e., % of energy for energy-providing nutrients and amount per 10 MJ of energy for foods and other nutrients).

### Assessment of overall diet quality

As measures of overall diet quality, the healthy diet indicator (HDI) and the Mediterranean diet score (MDS) were calculated, as described elsewhere [10]. Both diet quality measures have been shown to be prospectively associated with certain health outcomes such as increased survival and decreased disease burden [43–45]. The HDI was selected as a diet quality measure mainly based on nutrient intake while the MDS mainly based on food intake. The HDI includes six nutrients and one food group (saturated fat, polyunsaturated fat, cholesterol, protein, dietary fiber, fruits and vegetables, and non-milk extrinsic sugar) [43, 44]. When intake was within the recommended range according to WHO guidelines, a score of one was assigned to that component; otherwise, a score of zero was assigned, with a total score ranging from zero to seven. Hence, a higher score reflected a healthier dietary pattern. The MDS represents a Mediterranean-type diet and is based on the consumption of nine different components (vegetables; legumes; fruits, nuts, and seeds; cereals; fish; the ratio of unsaturated to saturated fats; meat; dairy products; and alcohol) [44, 45]. A score of one was assigned to moderate alcohol intake or, depending on the component, intake above or below the sex-specific median. Scores for all nine components were summed and resulted in a total range from zero to nine, whereby a higher score reflected better adherence to a Mediterranean-type diet.

### Calculation of Food Standards Agency nutrient profiling system diet score

A detailed description of the procedure for calculating FSA nutrient profiling system diet score has been published elsewhere [35–37]. Briefly, the FSA score was computed for each food and beverage on the basis of the nutrient content per 100 g. Positive points (0 to +10) are allocated for the content of energy (kJ), SFA (g), total sugar (g), and sodium (mg), while negative points (0 to −5) are allocated for the content of fruits/vegetables/nuts (g), dietary fiber (g), and protein (g). Scores for foods and beverages are thus based on a discrete continuous scale theoretically ranging from −15 (most healthy) to +40 (least healthy).

Aggregated scores at the individual level (FSA diet scores) were computed with the use of arithmetic EI-weighted means; for each food or beverage consumed, the FSA score of the food or beverage was multiplied by the EI provided by that food or beverage, and then these terms were summed for all foods and beverages consumed and divided by the sum of the EI provided by all foods and beverages for each individual. Thus, FSA scores of total diet, meals, and snacks were calculated as follows.

$$ \mathrm{FSA}\ \mathrm{score}\  \mathrm{of}\  \mathrm{total}\  \mathrm{diet}=\Sigma \left[\left(\mathrm{FSA}\ \mathrm{score}\  \mathrm{of}\  \mathrm{food}\right)\times \left(\mathrm{EI}\ \mathrm{from}\  \mathrm{food}\right)\right]/\left(\mathrm{total}\ \mathrm{EI}\right) $$

$$ \mathrm{FSA}\ \mathrm{score}\  \mathrm{of}\  \mathrm{meals}=\Sigma \left[\left(\mathrm{FSA}\ \mathrm{score}\  \mathrm{of}\  \mathrm{food}\  \mathrm{consumed}\ \mathrm{as}\ \mathrm{meals}\right)\times \left(\mathrm{EI}\ \mathrm{from}\  \mathrm{food}\  \mathrm{consumed}\ \mathrm{as}\ \mathrm{meals}\right)\right]/\left(\mathrm{EI}\ \mathrm{from}\  \mathrm{meals}\right) $$

$$ \mathrm{FSA}\ \mathrm{score}\  \mathrm{of}\  \mathrm{snacks}=\Sigma \left[\left(\mathrm{FSA}\ \mathrm{score}\  \mathrm{of}\  \mathrm{food}\  \mathrm{consumed}\ \mathrm{as}\ \mathrm{snacks}\right)\times \left(\mathrm{EI}\ \mathrm{from}\  \mathrm{food}\  \mathrm{consumed}\ \mathrm{as}\ \mathrm{snacks}\right)\right]/\left(\mathrm{EI}\ \mathrm{from}\  \mathrm{snacks}\right) $$

Alcoholic beverages were excluded from the computation, because they were excluded from the FSA scoring system. Increasing FSA diet score therefore reflected decreasing quality in the foods consumed.

### Definition of meals and snacks

In the present study, eating occasions were defined as any occasion when any food or drink was consumed (without considering energy content) [10, 13, 14, 20, 30]. If two eating occasions occurred in ≤15 min, the two events were counted as a single eating occasion; when >15 min separated two eating occasions, these were considered distinct eating occasions [10, 20, 21, 23, 30]. All eating occasions were further divided into either meals or snacks with the use of two different published definitions: on the basis of (1) clock time [24] and (2) contribution to total EI [25]. For the first definition, meals were defined as eating events reported during selected times of the day, that is, 0600–1000, 1200–1500, and 1800–2100 h [10]. All other eating occasions were considered snacks. For the second definition, a meal was defined as any eating episode comprising ≥15% of total EI, regardless of the time of day or composition of foods or beverages consumed [10, 46]. All other eating episodes were classified as a snack. For each participant, dietary intakes from meals and snacks were calculated. Also, eating frequency and meal and snack frequency were calculated based on all eating occasions except for those providing <210 kJ of energy [9, 10, 13, 14, 20, 30, 46]. It should be noted that no self-definition of eating occasions was included in the NDNS dietary record.

### Assessment of non-dietary variables

The socio-economic status of each respondent (i.e., occupational social class) was self-reported and categorized as manual or non-manual. Smoking status (never, former, or current) was also self-reported. A 7-d physical activity diary was carried out concurrently with the dietary record. A detailed description of the procedure has been published elsewhere [39, 40]. Briefly, the subject was shown by trained interviewers how to record the information, and was asked to record, to the nearest 10 min, how long they spent doing various activities on that day. Trained interviewers checked the completeness of records at least twice during the recording period. Subsequently, time spent daily in sleep, light, moderate, and vigorous-intensity activities was computed for each day of recording. The number of hours spent per day on each activity was multiplied by the metabolic equivalent (MET) value of that activity (derived from a published table) [47], and all MET-h products were summed to produce a total MET-h score for the day. A mean daily value over 7-d was used in the analysis. Overall dietary reporting status was evaluated based on the ratio of reported EI to estimated energy requirement (EER) [40]. We calculated each subject’s EER based on the information on age, weight, height, and physical activity, with the use of equations published in the US Dietary Reference Intakes [48]. Physical activity category was determined for each subject based on the physical activity level calculated as total MET-h/d (from the 7-d physical activity diary) divided by 24.

### Analytic sample

Of 3704 potentially eligible people identified for the study, 2251 (61%) participated in the survey. For the present analysis, we excluded a total of 736 subjects with missing information on the variables used (*n* 468 for anthropometric data; *n* 527 for dietary data; *n* 56 for social class; *n* 3 for smoking status; and *n* 593 for physical activity; some subjects had more than one missing value). We further excluded 28 underweight subjects (BMI <18.5 kg/m^2^) [49] and 36 subjects without any snacking occasion (based on either definition) during the 7-d period. The final analysis sample comprised 1451 adults aged 19–64 years (659 men and 792 women; 39% of eligible sample). Further exclusion of subjects who reported dieting or that illness had affected their eating during the diet recording period (*n* 386) did not alter the findings of the present study (data not shown). Although both dieting and illness are likely to have some influence on the quantity of diet, it is unknown whether or how these factors are associated with the quality of diet (i.e., energy-adjusted dietary intakes as well as overall diet quality). To minimize the possibility of bias caused by excluding these subjects, they were included in the analysis.

### Statistical analysis

All statistical analyses were performed for men and women separately, using SAS statistical software (version 9.4, SAS Institute Inc., Cary, North Carolina). Differences between intakes from meals and snacks were examined by the paired *t*-test. Associations between the FSA scores of meals and snacks (as well as the FSA score of total diet) and overall dietary intakes and quality (assessed by HDI and MDS) were investigated by linear regression analyses using the PROC REG procedure, with adjustment for age and social class. Both the FSA score of meals and that of snacks based on the same definition were entered simultaneously into the regression model. Linear regression analyses (using the PROC REG procedure) were also performed to explore the associations between the FSA scores of meals and snacks (as well as the FSA score of total diet) and BMI and WC. Potential confounding factors considered were age, social class, smoking status, physical activity, and meal and snack frequency (model 1). We further included EI:EER as a potential confounding factor (model 2). These potential confounding factors were selected a priori based on a comprehensive literature review of epidemiologic studies on meal and snack eating behaviors in relation to measures of body fatness [7, 8, 10, 12–14, 19–23, 25, 27, 30]. The FSA scores of meals and snacks were analyzed continuously after confirming the linearity of relations using tertile categories.

Data were not weighted to take account of known socio-demographic differences between responders and non-responders, not only because the impact of this adjustment for nutritional variables, applied as a weighting factor, was extremely small and not significant [39] but also because we were only interested in relationships between variables, rather than estimates of prevalence [30, 31, 40]. All reported *P* values are 2-tailed, and *P* values of <0.05 were considered statistically significant.

## Results

### Characteristics of subjects

The mean values of BMI and WC were, respectively, 27.3 kg/m^2^ and 96.0 cm in men and 26.8 kg/m^2^ and 83.1 cm in women (Table 1). In comparison with EER, reported EI was on average under-reported by 27% in men and 31% in women. Mean eating frequency was 5.65 times/d in men and 4.89 times/d in women. Meal and snack frequencies based on time were, respectively, 3.64 and 2.01 times/d in men and 3.30 and 1.60 times/d in women, while meal and snack frequencies based on EI contribution were, respectively, 2.29 and 3.35 times/d in men and 2.29 and 2.61 times/d in women.

**Table 1:** Characteristics of subjects

	Men (*n* 659)		Women (*n* 792)	
	Mean	SD	Mean	SD
Age (years)	42.5	11.9	42.4	12.0
Social class (%)				
Manual	45.7		32.2	
Non-manual	54.3		67.8	
Smoking status (%)				
Never	44.3		46.5	
Former	25.6		22.0	
Current	30.1		31.6	
Physical activity (MET-h/d)	46.0	10.0	42.3	4.1
BMI (kg/m^2^)	27.3	4.4	26.8	5.6
WC (cm)	96.0	11.1	83.1	11.9
EI:EER	0.73	0.19	0.69	0.18
Total EI (kJ/d)	9882	2510	6980	1744
Eating frequency (times/d)	5.65	1.88	4.89	1.41
MF_time_ (times/d)*	3.64	1.19	3.30	0.97
SF_time_ (times/d)*	2.01	1.12	1.60	0.84
MF_energy%_ (times/d)†	2.29	0.55	2.29	0.57
SF_energy%_ (times/d)†	3.35	2.03	2.61	1.53
Food intake from meals and snacks (g/10 MJ)				
Fruits, vegetables and nuts	249.5	173.5	357.5	247.9
Cereals	242.0	102.2	241.1	103.0
Biscuits, cakes and pastries	38.6	39.5	41.7	38.3
Fish	34.6	41.6	44.7	58.7
Meat	203.5	95.4	187.1	113.4
Dairy products	285.5	186.9	366.7	221.7
Soft drinks	127.9	205.5	128.4	217.0
Sugar, preserves and confectionery	33.7	34.3	31.7	34.4
Nutrient intake from meals and snacks				
Protein (% of energy)	15.3	2.8	15.9	3.3
Fat (% of energy)	33.5	5.8	33.6	6.5
Saturated fatty acid (% of energy)	12.6	3.0	12.7	3.3
Carbohydrate (% of energy)	44.8	7.0	46.6	7.1
Starch (% of energy)	25.5	5.7	26.4	5.7
Total sugar (% of energy)	19.3	6.3	20.2	6.5
Non-milk extrinsic sugar (% of energy)	12.6	6.0	11.5	6.1
Alcohol (% of energy)	6.5	7.0	4.1	5.5
Dietary fiber (g/10 MJ)	16.0	5.4	18.7	7.0
Sodium (mg/10 MJ)	3397	709	3414	833
Diet quality score				
Healthy diet indicator‡	2.27	1.36	2.50	1.34
Mediterranean diet score§	4.43	1.69	4.39	1.67

MET, metabolic equivalent; BMI, body mass index; WC, waist circumference; EI:EER, ratio of energy intake to estimated energy requirement; EI, energy intake; MF_time_, meal frequency (MF) determined based on the time consumed; SF_time_, snack frequency (SF) determined based on the time consumed; MF_energy%_, MF determined based on percentage contribution to total EI; SF_energy%_, SF determined based on percentage contribution to total EI

*Meals were defined as eating events reported during select times of the day (0600–1000, 1200–1500, and 1800–2100 h); all other eating occasions were considered as snacks

†A meal was defined as any eating episode comprising ≥15% of total EI, regardless of the time of day or composition of foods and beverages consumed; all other eating episodes were classified as snacks

‡Possible score ranging from 0 to 7. A higher score reflecting a better diet quality

§Possible score ranging from 0 to 9. A higher score reflecting a better diet quality

### Characteristics of meals and snacks

Characteristics of meals and snacks were generally similar in men and women (Table 2). Irrespective of the definitions of meals and snacks, EI from meals was larger than that from snacks, contributing to, on average, 64% to 84% of total EI. In terms of dietary components for the FSA score calculation, compared with snacks, meals were higher in energy, sodium, dietary fiber, and protein and lower in total sugar. Meals were also higher in SFA and lower in fruits/vegetables/nuts than snacks in both sexes when meals and snacks were defined based on EI contribution. Similar differences in SFA and fruits/vegetables/nuts were observed only in women when meals and snacks were defined based on time. Consequently, meals were lower in the FSA score, reflecting a better nutritional quality than snacks in both sexes when the time-based meals and snacks were compared. When meals and snacks based on EI contribution were examined, a better quality of meals compared with snacks was observed only in women.

**Table 2:** Characteristics of meals and snacks

			Meals and snacks based on time*					Meals and snacks based on EI contribution^†^				
	Total intake		Intake from meals		Intake from snacks			Intake from meals		Intake from snacks		
	Mean	SD	Mean	SD	Mean	SD	*P* ^‡^	Mean	SD	Mean	SD	*P* ^‡^
Men (*n* 659)												
Energy (kJ/d)	9882	2510	6960	2249	2922	1727	<0.0001	7457	1900	2425	1536	<0.0001
SFA (g/10 MJ)	33.3	7.9	33.1	8.5	33.2	11.8	0.76	35.8	8.2	26.3	13.7	<0.0001
Total sugar (g/10 MJ)	122.0	39.5	118.3	43.0	148.1	72.2	<0.0001	84.5	32.5	247.4	95.1	<0.0001
Sodium (mg/10 MJ)	3397	709	3495	817	3001	1321	<0.0001	3838	792	2077	1270	<0.0001
Dietary fiber (g/10 MJ)	23.5	12.2	16.6	5.9	13.8	7.4	<0.0001	17.6	5.2	11.2	9.0	<0.0001
Protein (g/10 MJ)	90.8	16.6	93.4	18.2	80.6	27.6	<0.0001	101.6	18.6	59.5	26.5	<0.0001
Fruits, vegetables and nuts (g/10 MJ)	249.5	173.5	253.8	188.9	246.7	289.1	0.51	250.3	170.9	286.4	420.1	0.02
FSA score§	5.55	2.04	5.44	2.20	6.02	3.25	<0.0001	5.54	2.03	5.52	3.52	0.87
Women (*n* 792)												
Energy (kJ/d)	6980	1744	4992	1581	1987	1195	<0.0001	5395	1342	1584	914	<0.0001
SFA (g/10 MJ)	33.6	8.8	33.2	9.4	34.3	13.4	0.01	35.0	9.1	29.0	14.1	<0.0001
Total sugar (g/10 MJ)	127.8	4.1	124.4	47.5	160.0	82.8	<0.0001	92.7	33.4	257.5	88.8	<0.0001
Sodium (mg/10 MJ)	3414	833	3539	899	2885	1281	<0.0001	3726	885	2326	1517	<0.0001
Dietary fiber (g/10 MJ)	29.8	17.2	19.3	8.2	16.3	8.8	<0.0001	19.8	6.7	15.1	11.5	<0.0001
Protein (g/10 MJ)	94.3	19.3	95.2	21.5	85.5	32.0	<0.0001	102.2	20.5	67.4	27.3	<0.0001
Fruits, vegetables and nuts (g/10 MJ)	357.5	247.9	353.1	259.1	388.7	460.6	0.02	336.0	225.4	483.9	610.9	<0.0001
FSA score§	5.24	2.16	5.15	2.38	5.88	3.51	<0.0001	5.14	2.16	5.37	3.59	0.0499

SFA, saturated fatty acid; FSA, Food Standards Agency

*Meals were defined as eating events reported during select times of the day (0600–1000, 1200–1500, and 1800–2100 h); all other eating occasions were considered as snacks

^†^A meal was defined as any eating episode comprising ≥15% of total energy intake, regardless of the time of day or composition of foods and beverages consumed; all other eating episodes were classified as snacks

^‡^ *P* values for differences between meal and snack based on the paired *t*-test

§Possible score ranging from −15 to 40. A lower score reflecting a better nutritional quality

### Associations of Food Standards Agency nutrient profiling system scores of meals and snacks with total dietary intakes

The associations of the FSA scores of meals and snacks (as well as the FSA score of total diet) with total dietary intakes were generally similar in both men (Table 3) and women (Table 4). Irrespective of the definition of meals and snacks, higher FSA scores (lower nutritional quality) of meals and snacks (and total diet) were associated with unfavorable profiles of individual components of the diet, including lower intakes of fruits/vegetables/nuts and higher intakes of biscuits/cakes/pastries, total fat, and SFA. The FSA score of meals (and total diet) was also inversely associated with intakes of cereals, fish, meat, dairy products, protein, starch, and dietary fiber and positively associated with intakes of sugar/preserves/confectionery and energy. The FSA score of snacks (and total diet) was inversely associated with alcohol intake and positively with non-milk extrinsic sugar intake. There was no association with sodium intake (except for an inverse association for the EI-contribution-based snacks in women). The associations were inconsistent for soft drinks, carbohydrate, and total sugar. However, the strength of the associations was generally stronger for meals compared with snacks, which was clearly shown in the analysis of measures of overall diet quality. Irrespective of the definition of meals and snacks, one-point increase of the FSA score of meals was associated with 0.17–0.22 point decrease of HDI and 0.19–0.25 point decrease of MDS, while one-point increase of the FSA score of snacks was associated with only 0.03–0.06 point decrease of HDI and 0.05–0.08 decrease of MDS (all *P* ≤ 0.005).

**Table 3:** Associations of FSA scores of meals and snacks with total dietary intakes in men (*n* 659)*

	Meals and snacks based on time^†^						Meals and snacks based on EI contribution^‡^						FSA score of meals and snacks		
	FSA score of meals			FSA score of snacks			FSA score of meals			FSA score of snacks					
	β^§^	95% CI^§^	*P*	β^§^	95% CI^§^	*P*	β^§^	95% CI^§^	*P*	β^§^	95% CI^§^	*P*	β^§^	95% CI^§^	*P*
Food intake from meals and snacks (g/10 MJ)															
Fruits, vegetables and nuts	−25.63	−31.37, −19.90	<0.0001	−9.06	−12.84, −5.28	<0.0001	−34.65	−40.62, −28.68	<0.0001	−4.93	−8.31, −1.54	0.003	−37.64	−43.16, −32.12	<0.0001
Cereals	−11.26	−15.15, −7.37	<0.0001	−0.99	−3.56, 1.57	0.45	−15.29	−19.35, −11.23	<0.0001	0.09	−2.21, 2.39	0.94	−13.63	−17.38, −9.87	<0.0001
Biscuits, cakes and pastries	4.66	3.24, 6.07	<0.0001	2.64	1.71, 3.58	<0.0001	5.24	3.73, 6.75	<0.0001	2.20	1.35, 3.05	<0.0001	7.58	6.20, 8.96	<0.0001
Fish	−3.60	−5.17, −2.03	<0.0001	−1.04	−2.08, −0.009	0.048	−4.65	−6.31, −2.99	<0.0001	−0.13	−1.07, 0.81	0.79	−4.92	−6.44, −3.40	<0.0001
Meat	−5.71	−9.40, −2.02	0.003	−2.56	−4.99, −0.12	0.04	−8.18	−12.07, −4.30	<0.0001	−1.85	−4.05, 0.35	0.10	−9.10	−12.67, −5.52	<0.0001
Dairy products	−11.86	−19.15, −4.57	0.002	2.60	−2.21, 7.41	0.29	−9.64	−17.36, −1.91	0.01	−0.28	−4.66, 4.09	0.90	−8.12	−15.22, −1.02	0.03
Soft drinks	2.87	−4.86, 10.60	0.47	2.82	−2.27, 7.92	0.28	12.90	4.78, 21.03	0.002	−4.12	−8.72, 0.49	0.08	7.22	−0.28, 14.71	0.06
Sugar, preserves and confectionery	5.23	4.01, 6.44	<0.0001	1.84	−0.11, 0.29	0.38	2.33	1.10, 3.57	0.0002	4.21	3.51, 4.91	<0.0001	7.69	6.52, 8.86	<0.0001
Nutrient intake from meals and snacks															
Protein (% of energy)	−0.51	−0.61, −0.41	<0.0001	−0.09	−0.16, −0.03	0.007	−0.61	−0.72, −0.51	<0.0001	−0.05	−0.11, 0.01	0.08	−0.63	−0.72, −0.53	<0.0001
Fat (% of energy)	1.05	0.85, 1.24	<0.0001	0.45	0.33, 0.58	<0.0001	1.46	1.26, 1.66	<0.0001	0.24	0.13, 0.35	<0.0001	1.65	1.47, 1.84	<0.0001
Saturated fatty acid (% of energy)	0.67	0.58, 0.76	<0.0001	0.29	0.23, 0.34	<0.0001	0.87	0.78, 0.96	<0.0001	0.19	0.14, 0.24	<0.0001	1.05	0.97, 1.14	<0.0001
Carbohydrate (% of energy)	−0.19	−0.47, 0.08	0.17	0.14	−0.04, 0.32	0.14	−0.97	−1.25, −0.69	<0.0001	0.56	0.40, 0.72	<0.0001	−0.11	−0.38, 0.16	0.44
Starch (% of energy)	−0.44	−0.67, −0.22	<0.0001	0.01	−0.14, 0.16	0.90	−0.75	−0.98, −0.52	<0.0001	0.12	−0.01, 0.25	0.07	−0.50	−0.72, −0.29	<0.0001
Total sugar (g/10 MJ)	0.25	0.004, 0.50	0.047	0.13	−0.03, 0.29	0.12	−0.21	−0.47, 0.04	0.10	0.44	0.29, 0.58	<0.0001	0.40	0.16, 0.64	0.001
Non-milk extrinsic sugar (% of energy)	0.68	0.45, 0.90	<0.0001	0.18	0.04, 0.33	0.01	0.30	0.06, 0.53	0.01	0.48	0.35, 0.61	<0.0001	0.90	0.68, 1.12	<0.0001
Alcohol (% of energy)	−0.35	−0.61, −0.09	0.009	−0.51	−0.68, −0.33	<0.0001	0.12	−0.16, 0.39	0.40	−0.75	−0.91, −0.60	<0.0001	−0.93	−1.19, −0.67	<0.0001
Dietary fiber (g/10 MJ)	−0.95	−1.13, −0.76	<0.0001	−0.15	−0.27, −0.03	0.02	−1.27	−1.46, −1.08	<0.0001	0.003	−0.11, 0.11	0.95	−1.17	−1.35, −0.99	<0.0001
Sodium (g/10 MJ)	20.05	−7.94, 48.04	0.16	−8.11	−26.58, 10.35	0.39	19.46	−10.14, 49.06	0.20	−5.160	−21.93, 11.61	0.55	11.89	−15.30, 39.08	0.39
Total EI (kJ/d)	152.3	55.7, 248.9	0.002	111.0	47.2, 174.7	0.0007	281.7	179.7, 383.6	<0.0001	29.2	−28.6, 86.9	0.32	300.1	206.5, 393.7	<0.0001
Diet quality score															
Healthy diet indicator^||^	−0.19	−0.24, −0.14	<0.0001	−0.05	−0.09, −0.02	0.002	−0.20	−0.26, −0.15	<0.0001	−0.06	−0.09, −0.03	<0.0001	−0.27	−0.32, −0.22	<0.0001
Mediterranean diet score^¶^	−0.19	−0.25, −0.13	<0.0001	−0.08	−0.12, −0.04	0.0002	−0.25	−0.32, −0.19	<0.0001	−0.05	−0.09, −0.02	0.005	−0.30	−0.36, −0.24	<0.0001

FSA, Food Standards Agency; EI, energy intake

*Adjustment was made for age (years, continuous) and social class (manual or non-manual). Both FSA score of meals and FSA score of snacks based on the same definition were entered simultaneously into the regression model. FSA scores ranging from −15 to 40, with a lower score reflecting a better nutritional quality

^†^Meals were defined as eating events reported during select times of the day (0600–1000, 1200–1500, and 1800–2100 h); all other eating occasions were considered as snacks

^‡^A meal was defined as any eating episode comprising ≥15% of total EI, regardless of the time of day or composition of foods and beverages consumed; all other eating episodes were classified as snacks

^§^Regression coefficients mean the change of dietary variables with 1-unit increase of FSA diet score

^||^Possible score ranging from 0 to 7. A higher score reflecting a better diet quality

^¶^Possible score ranging from 0 to 9. A higher score reflecting a better diet quality

**Table 4:** Associations of FSA scores of meals and snacks with total dietary intakes in women (*n* 792)*

	Meals and snacks based on time†						Meals and snacks based on EI contribution‡						FSA score of meals and snacks		
	FSA score of meals			FSA score of snacks			FSA score of meals			FSA score of snacks					
	β§	95% CI§	*P*	β§	95% CI§	*P*	β§	95% CI§	*P*	β§	95% CI§	*P*	β§	95% CI§	*P*
Food intake from meals and snacks (g/10 MJ)															
Fruits, vegetables and nuts	−44.11	−50.49, −37.72	<0.0001	−10.10	−14.39, −5.81	<0.0001	−40.62	−47.88, −33.37	<0.0001	−17.30	−21.65, −12.95	<0.0001	−57.96	−64.49, −51.43	<0.0001
Cereals	−9.10	−12.25, −5.94	<0.0001	−1.49	−3.61, 0.63	0.17	−10.99	−14.64, −7.34	<0.0001	−1.00	−3.19, 1.19	0.37	−12.01	−15.26, −8.76	<0.0001
Biscuits, cakes and pastries	3.63	2.53, 4.73	<0.0001	2.20	1.46, 2.94	<0.0001	3.30	2.03, 4.56	<0.0001	2.70	1.94, 3.46	<0.0001	6.31	5.17, 7.45	<0.0001
Fish	−5.65	−7.38, −3.92	<0.0001	−2.17	−3.33, −1.01	0.0003	−6.82	−8.81, −4.83	<0.0001	−1.80	−3.00, −0.61	0.003	−8.46	−10.24, −6.69	<0.0001
Meat	−4.80	−8.31, −1.30	0.007	−2.01	−4.36, 0.35	0.09	−6.63	−10.70, −2.56	0.001	−0.76	−3.20, 1.68	0.54	−6.95	−10.58, −3.32	0.0002
Dairy products	−16.74	−23.40, −10.07	<0.0001	−6.71	−11.19, −2.23	0.003	−10.61	−18.35, −2.88	0.007	−10.70	−15.34, −6.05	<0.0001	−22.73	−29.66, −15.81	<0.0001
Soft drinks	7.87	1.30, 14.44	0.02	−0.36	−4.78, 4.05	0.87	3.52	−4.11, 11.16	0.37	2.65	−1.94, 7.23	0.26	6.22	−0.59, 13.03	0.07
Sugar, preserves and confectionery	5.19	4.22, 6.15	<0.0001	1.70	1.05, 2.34	<0.0001	1.82	0.74, 2.90	0.001	4.21	3.56, 4.85	<0.0001	7.07	6.08, 8.07	<0.0001
Nutrient intake from meals and snacks															
Protein (% of energy)	−0.54	−0.62, −0.45	<0.0001	−0.20	−0.25, −0.14	<0.0001	−0.51	−0.61, −0.41	<0.0001	−0.23	−0.29, −0.18	<0.0001	−0.76	−0.85, −0.67	<0.0001
Fat (% of energy)	1.36	1.20, 1.53	<0.0001	0.42	0.31, 0.53	<0.0001	1.83	1.65, 2.01	<0.0001	0.16	0.05, 0.27	0.005	1.93	1.77, 2.10	<0.0001
Saturated fatty acid (% of energy)	0.74	0.66, 0.82	<0.0001	0.25	0.20, 0.30	<0.0001	1.04	0.96, 1.13	<0.0001	0.07	0.02, 0.12	0.009	1.07	0.99, 1.15	<0.0001
Carbohydrate (% of energy)	−0.44	−0.66, −0.22	<0.0001	−0.03	−0.17, 0.12	0.74	−1.22	−1.47, −0.98	<0.0001	0.47	0.32, 0.61	<0.0001	−0.57	−0.80, −0.35	<0.0001
Starch (% of energy)	−0.39	−0.56, −0.21	<0.0001	−0.07	−0.19, 0.05	0.24	−0.77	−0.97, −0.57	<0.0001	0.19	0.07, 0.31	0.002	−0.53	−0.72, −0.35	<0.0001
Total sugar (g/10 MJ)	−0.05	−0.26, 0.15	0.60	0.04	−0.09, 0.18	0.52	−0.46	−0.69, −0.22	0.0001	0.27	0.13, 0.41	0.0001	−0.04	−0.25, 0.17	0.71
Non-milk extrinsic sugar (% of energy)	0.66	0.48, 0.83	<0.0001	0.19	0.07, 0.31	0.002	0.13	−0.07, 0.33	0.21	0.57	0.45, 0.70	<0.0001	0.86	0.67, 1.04	<0.0001
Alcohol (% of energy)	−0.39	−0.55, −0.22	<0.0001	−0.21	−0.32, −0.09	0.0003	−0.10	−0.29, 0.10	0.33	−0.39	−0.51, −0.28	<0.0001	−0.60	−0.78, −0.43	<0.0001
Dietary fiber (g/10 MJ)	−1.40	−1.58, −1.22	<0.0001	−0.22	−0.34, −0.10	0.0004	−1.59	−1.80, −1.39	<0.0001	−0.22	−0.34, −0.09	0.0005	−1.77	−1.95, −1.58	<0.0001
Sodium (g/10 MJ)	−6.28	−32.36, 19.80	0.64	−7.51	−25.03, 10.02	0.40	27.76	−2.33, 57.86	0.07	−30.46	−48.52, −12.39	0.001	−11.29	−38.30, 15.72	0.41
Total EI (kJ/d)	125.1	73.6, 176.7	<0.0001	104.10	69.4, 138.7	<0.0001	130.4	71.3. 189.6	<0.0001	116.7	81.2, 152.2	<0.0001	258.2	205.0, 311.5	<0.0001
Diet quality score															
Healthy diet indicator||	−0.17	−0.21, −0.13	<0.0001	−0.05	−0.08, −0.03	0.0001	−0.22	−0.26, −0.18	<0.0001	−0.03	−0.06, −0.01	0.02	−0.24	−0.28, −0.20	<0.0001
Mediterranean diet score¶	−0.20	−0.25, −0.15	<0.0001	−0.05	−0.09, −0.02	0.002	−0.24	−0.29, −0.18	<0.0001	−0.05	−0.08, −0.01	0.005	−0.28	−0.33, −0.23	<0.0001

FSA, Food Standards Agency; EI, energy intake

*Adjustment was made for age (years, continuous) and social class (manual or non-manual). Both FSA score of meals and FSA score of snacks based on the same definition were entered simultaneously into the regression model. FSA scores ranging from −15 to 40, with a lower score reflecting a better nutritional quality

†Meals were defined as eating events reported during select times of the day (0600–1000, 1200–1500, and 1800–2100 h); all other eating occasions were considered as snacks

‡A meal was defined as any eating episode comprising ≥15% of total EI, regardless of the time of day or composition of foods and beverages consumed; all other eating episodes were classified as snacks

§Regression coefficients mean the change of dietary variables with 1-unit increase of FSA diet score

|| Possible score ranging from 0 to 7. A higher score reflecting a better diet quality

¶Possible score ranging from 0 to 9. A higher score reflecting a better diet quality

### Associations of Food Standards Agency nutrient profiling system scores of meals and snacks with measures of body fatness

After adjustment for potential confounding factors (i.e., age, social class, smoking status, physical activity, and meal and snack frequency; model 1), the FSA score of meals based on EI contribution was inversely associated with BMI and WC in only women, while the FSA score of total diet was inversely associated with WC in only men (Table 5). However, these inverse associations were not observed with further adjustment for EI:EER (model 2). Instead, the analysis showed positive associations between the FSA score of snacks based on EI contribution and BMI and WC in only women. There were no associations for the FSA scores of meals and snacks based on time in any analysis.

**Table 5:** Associations of FSA scores of meals and snacks with measures of body fatness*

	Men (*n* 659)						Women (*n* 792)					
	Model 1†			Model 2‡			Model 1†			Model 2‡		
	β§	95% CI§	*P*	β§	95% CI§	*P*	β§	95% CI§	*P*	β§	95% CI§	*P*
FSA score of meals based on time||												
BMI (kg/m^2^)	0.03	−0.14, 0.20	0.76	0.07	−0.09, 0.24	0.39	−0.17	−0.35, 0.01	0.06	−0.02	−0.20, 0.15	0.80
WC (cm)	0.20	−0.22, 0.62	0.34	0.30	−0.11, 0.72	0.15	−0.21	−0.58, 0.16	0.26	0.04	−0.32, 0.41	0.82
FSA score of snacks based on time||												
BMI (kg/m^2^)	−0.11	−0.22, 0.003	0.06	−0.06	−0.17, 0.05	0.28	0.04	−0.08, 0.16	0.52	0.11	−0.01, 0.22	0.07
WC (cm)	−0.12	−0.39, 0.15	0.39	−0.02	−0.29, 0.25	0.91	−0.06	−0.30, 0.19	0.65	0.06	−0.18, 0.30	0.62
FSA score of meals based on EI contribution¶												
BMI (kg/m^2^)	0.02	−0.16, 0.19	0.87	0.12	−0.06, 0.30	0.19	−0.24	−0.45, −0.04	0.02	−0.07	−0.27, 0.13	0.48
WC (cm)	0.11	−0.33, 0.55	0.63	0.33	−0.12, 0.77	0.15	−0.48	−0.91, −0.05	0.03	−0.18	−0.60, 0.25	0.41
FSA score of snacks based on EI contribution¶												
BMI (kg/m^2^)	−0.08	−0.18, 0.02	0.12	−0.08	−0.18, 0.02	0.11	0.11	−0.01, 0.23	0.07	0.17	0.05, 0.29	0.005
WC (cm)	−0.02	−0.27, 0.23	0.87	−0.02	−0.27, 0.22	0.85	0.24	−0.01, 0.50	0.06	0.34	0.09, 0.59	0.007
FSA score of meals and snacks												
BMI (kg/m^2^)	−0.06	−0.22, 0.11	0.50	0.05	−0.11, 0.22	0.55	−0.11	−0.29, 0.07	0.23	0.12	−0.06, 0.30	0.20
WC (cm)	−0.23	−0.40, −0.05	0.01	0.10	−0.10, 0.29	0.35	−0.21	−0.59, 0.17	0.27	0.19	−0.20, 0.58	0.33

FSA, Food Standards Agency; BMI, body mass index; WC, waist circumference

*FSA scores ranging from −15 to 40, with a lower score reflecting a better nutritional quality

†Adjusted for age (years, continuous), social class (manual or non-manual), smoking status (never, former, or current), physical activity (metabolic equivalent-h/d, continuous), and meal frequency (times/d, continuous) and snack frequency (times/d, continuous) based on the same definition (or the sum of meal and snack frequency (eating frequency; times/d, continuous) in the case of FSA diet score of meals and snacks). Both FSA score of meals and FSA score of snacks based on the same definition were entered simultaneously into the regression model

‡Adjusted for variables used in model 1 and ratio of reported energy intake to estimated energy requirement (continuous). Both FSA score of meals and FSA score of snacks based on the same definition were entered simultaneously into the regression model

§Regression coefficients mean the change of adiposity measures with 1-unit increase of FSA diet score

|| Meals were defined as eating events reported during select times of the day (0600–1000, 1200–1500, and 1800–2100 h); all other eating occasions were considered as snacks

¶A meal was defined as any eating episode comprising ≥15% of total EI, regardless of the time of day or composition of foods and beverages consumed; all other eating episodes were classified as snacks

## Discussion

To our knowledge, this is the first study to examine how the nutritional quality of meals and snacks is associated with overall diet quality and measures of body fatness. Irrespective of the definition of meals and snacks, lower nutritional quality of both meals and snacks (assessed by the FSA score) was associated with lower quality of overall diet (assessed by HDI and MDS) in British adults. However, the associations were stronger for meals, mainly due to their larger contribution to total EI. The associations with BMI and WC were not clear or consistent. The present findings suggest that the nutritional quality of meals is more strongly associated with overall diet quality (but not necessarily measures of body fatness) than the nutritional quality of snacks.

In the present study, 16% to 36% of EI was derived from snacks, depending on the definition of snacks and sex. These figures are within the range of those observed in other countries, including Norway (17% for men and 21% for women) [15], Brazil (21% for both sex combined) [24], the US (23% for both men and women) [16], and Finland (36% for men and 40% for women) [17]. This suggests that a considerable proportion of total EI is derived from snacks in affluent countries, whatever definitions are applied.

Only a very limited number of studies have compared the dietary composition of meals and snacks. Although the definitions of meals and snacks varied across studies, a consistent finding is that meals provide a higher proportion of EI from fat or protein than did snacks [18, 26–29]. Meals had a lower proportion of total sugars but not total carbohydrate [26] or had a higher density of dietary fiber than did snacks [27, 29]. These observations are generally similar to those obtained in the present study. Taken together, it is speculated that meals and snacks are differentially associated with overall diet quality and health status. In the present study, lower nutritional quality of meals (assessed by the FSA score) was associated with lower overall diet quality (assessed by HDI and MDS), independent of the definition of meals. Lower nutritional quality of snacks was similarly associated with lower overall diet quality, but the associations were generally weaker mainly due to their smaller contribution to total EI than meals. However, given some evidence that nutritional quality of snack is better in the morning compared to later in the day [50], future research considering each snack occasion separately according to the time of day would be of interest. In any case, because some of the components for calculating the FSA diet score (e.g., fruits, vegetables, nuts, SFA, protein, and dietary fiber) are also those for HDI and MDS, the present results should be interpreted with this kind of circularity between FSA score and HDI and MDS in mind. Nevertheless, this circularity should not explain the difference of the strengths of the associations.

For measures of body fatness, we did not observe clear or consistent associations with diet quality of meals and snacks, which may not be unexpected given that the FSA score does not necessarily focus on obesity prevention. The present study showed a number of sex differences in the associations of nutritional quality of meals and snacks with dietary intake and measures of body fatness, which might be due to sex differences in dietary habits (e.g., higher snack frequency, higher proportion of EI from snacks, and lower intakes of fruits/vegetables/nuts and dairy products in men). In any case, as a few existing studies on meal and snack eating behaviors in relation to overall diet and measures of body fatness have been producing somewhat conflicting findings [7–11], more research, preferably with a prospective design, should be conducted before reaching a firm conclusion.

The present study showed modest but significant associations of nutritional quality of meals and snacks assessed by the FSA score with overall diet quality. Theoretically, the FSA score could be used in any population with any dietary habit because the score is calculated based on dietary components per weight of foods and beverages. This is not the case for many established measures for assessing overall diet quality, because they are usually developed based on dietary guidelines targeted for specific populations. Additionally, because the FSA score is weighted for EI, the score can be directly compared between meals and snacks within populations in addition to among populations, which is not the case for, for example, MDS where scoring is dependent on the distribution of dietary intake within populations. Taken together, the FSA score might be a useful tool for assessing nutritional quality of meals and snacks in future research.

In the present study, the direction of the association of nutritional quality of meals and snacks with BMI and WC somewhat changed after adjustment for EI:EER. Given that under-reporters were characterized by higher BMI and WC and higher nutritional quality of meals and snacks (data not shown), this may be due to the under-reporting of energy-dense or less healthy foods (and hence over-estimation of diet quality) concomitant with the under-reporting of EI by subjects with higher BMI and WC [30]. Thus, the present study may highlight the key importance of adjusting for EI misreporting in studies of dietary variables associated with EI misreporting (the FSA diet score in this case) in relation to measures of body fatness.

The strengths of this study include the use of objective and published definitions of meals and snacks based on detailed dietary information obtained from a 7-d weighed dietary record, measured anthropometric data, and the use of individualized measure of EER for assessing EI misreporting. However, there are also several limitations. First, the cross-sectional nature of the study does not permit the assessment of causality owing to the uncertain temporality of the association. Only a prospective study would provide better understanding of the relation between meal and snack intake and overall diet quality and measures of body fatness.

Another limitation of the present study is the relatively low response rate (61%), and only 39% of the eligible sample was included in the present study. The subjects included in the present analysis (*n* 1451) differed somewhat from those excluded from the analysis (*n* 705–758 depending on variables). The excluded subjects were more likely to be younger, be in manual occupations, and be current smokers (all *P* < 0.05). However, a previous analysis concluded that there was no evidence to suggest serious non-response bias in NDNS [39]. Additionally, although pregnant and lactating women were excluded from the sample of NDNS, postpartum and non-lactating women were included in the present analysis (because of a lack of information), which might cause bias with regard to WC and BMI. Further, although we adjusted for a variety of potential confounding variables, residual confounding could not be ruled out.

Finally, because there is no definitive consensus about what constitutes a snack or a meal, the present results should be interpreted cautiously and oversimplification should be avoided. As mentioned above, we could not conduct the present analysis based on self-identification of eating occasions, the most common definition of meals and snacks (because of a lack of information in NDNS), although it is subject to inconsistencies due to differences in individual perception [32]. Additionally, meals and snacks based on time may be problematic, because eating patterns vary according to lifestyle (e.g., shift workers, individuals who consistently eat their meals at non-traditional times of day) as well as the cultural environment [32]. Furthermore, meals and snacks based on EI contribution (≥15% or <15%) was made on the basis of the US national averages of the distribution of energy from (self-defined) meals compared with (self-defined) snacks (breakfast: ≈ 16%; lunch: ≈ 25%; dinner: ≈ 37%; and snack: ≈ 22% from two occasions) [16]. This may not be suitable in the present British population, although the EI-contribution-based definition has recently been proposed for surveys where no information on self-identification of eating occasions is available [46]. Thus, results may possibly differ on the basis of other definitions. In any case, as research explicitly examining the impact of these different definitions is limited, further research using different definitions of meals and snacks is warranted.

## Conclusions

In this cross-sectional study in British adults, lower nutritional quality of meals (assessed by the FSA score) was associated with lower overall diet quality (assessed by HDI and MDS). Lower nutritional quality of snacks was similarly associated with lower overall diet quality, but the associations were generally weaker mainly due to their lower contribution to total EI. These associations were not dependent on the definition of meals and snacks. The associations between nutritional quality of meals and snacks and BMI and WC were not clear or consistent. The present findings suggest stronger associations of nutritional quality of meals with overall diet quality (but not necessarily measures of body fatness) than that of snacks. Further research, particularly with a prospective design, is needed so that any firm conclusions can be drawn with regard to the effect of different combinations of foods in meals and snacks on overall diet quality and measures of body fatness.

## Abbreviations

BMI: Body mass index
EER: Estimated energy requirement
EI: Energy intake
EI:EER: Ratio of energy intake to estimated energy requirement
FSA: Food Standards Agency
HDI: Healthy diet indicator
MDS: Mediterranean diet score
MET: Metabolic equivalent
NDNS: National Diet and Nutrition Survey
SFA: Saturated fatty acid
WC: Waist circumference
